# Cytotoxic Effect of Paclitaxel and Lapatinib *Co*-Delivered in Polylactide-*co*-Poly(ethylene glycol) Micelles on HER-2-Negative Breast Cancer Cells

**DOI:** 10.3390/pharmaceutics11040169

**Published:** 2019-04-06

**Authors:** Alicja Zajdel, Adam Wilczok, Katarzyna Jelonek, Monika Musiał-Kulik, Aleksander Foryś, Suming Li, Janusz Kasperczyk

**Affiliations:** 1 School of Pharmacy with the Division of Laboratory Medicine in Sosnowiec, Medical University of Silesia, Katowice, Poland, Department of Biopharmacy, Jedności 8, 41-200 Sosnowiec, Poland; awilczok@sum.edu.pl (A.W.); janusz.kasperczyk@sum.edu.pl (J.K.); 2 Centre of Polymer and Carbon Materials, Polish Academy of Sciences, Curie-Sklodowska 34 St., 41-819 Zabrze, Poland; kjelonek@cmpw-pan.edu.pl (K.J.); mmusial@cmpw-pan.edu.pl (M.M.-K.); aforys@cmpw-pan.edu.pl (A.F.); 3 European Institute of Membranes, UMR CNRS 5635, University of Montpellier, Place Eugene Bataillon, 34095 Montpellier CEDEX 5, France; suming.li@univ-montp2.fr

**Keywords:** PLA-PEG micelles, paclitaxel, lapatinib, MCF-7 breast cancer cells

## Abstract

To find better strategies to enhance the cytotoxic effect of paclitaxel (PTX) and lapatinib (LAP) against breast cancer cells, we analyzed the efficacy of a novel delivery system containing polylactide-co-poly(ethylene glycol) (PLA-PEG) filomicelles of over 100 nm in length and spherical micelles of approximately 20 nm in diameter. The ^1^H NMR measurements confirmed the incorporation of PTX and LAP into micelles. Analysis of the drug release mechanism revealed the diffusion-controlled release of LAP and anomalous transport of PTX. Drug content analysis in lyophilized micelles and micellar solution showed their good storage stability for at least 6 weeks. Blank micelles, LAP-loaded micelles and free LAP did not affect MCF-7 breast cancer cell proliferation, suggesting that the cytotoxicity of PTX-, PTX/LAP-loaded micelles, and the binary mixture of free PTX and LAP was solely caused by PTX. PTX/LAP-loaded micelles showed greater toxicity compared to the binary mixture of PTX and LAP after 48 h and 72 h. Only free PTX alone induced P-gp activity. This study showed the feasibility of using a LAP and PTX combination to overcome MDR in MCF-7 cells, particularly when co-loaded into micelles. We suggest that PTX/LAP micelles can be applicable not only for the therapy of HER-2-positive, but also HER-2-negative breast cancers.

## 1. Introduction

Despite significant progress in the last few decades in breast cancer treatment and the use of different therapeutic strategies, this cancer remains a serious clinical problem due to the nature of its proliferation and loss of sensitivity to pharmacological agents [1].

Paclitaxel (PTX) is used to treat breast cancer both as a monotherapy and in combination with other anticancer drugs depending on the severity of the cancer, the presence of metastases and preceding therapeutic management. It is characterized by high effectiveness both in early breast cancer and in metastatic breast cancer [2]. The primary or acquired multidrug resistance (MDR) of tumor cells to taxanes is a significant clinical problem in the treatment of various histological types of breast cancer. The main problem of MDR in tumor cells is the complexity and multifactorial nature of this phenomenon, which is conditioned by numerous different mechanisms that interact with each other. Among the known mechanisms of breast cancer cells resistance to PTX, the most important are the active removal of the drug from the cell related to the increased activity of ATP-binding cassette (ABC) membrane transporters such as P-glycoprotein (P-gp; ABCB1), multidrug resistance-associated proteins (MRPs; ABCC), and breast cancer resistance proteins (BCRP; ABCG2) [3,4], enhanced drug detoxification by cytochrome P450, CYP3A4/5 and CYP2C8 enzymes [5,6], alterations in the molecular targets, microtubule and disorders of microtubule-associated proteins (MAPs) [7,8], or apoptosis [9,10,11]. Tyrosine kinase inhibitors (TKIs) are known to sensitize tumor cells to anticancer drugs, e.g., PTX or doxorubicin. The ability to inhibit P-gp, MRPs, or BCRP may increase their efficacy when used in combined therapy [12,13]. Lapatinib (LAP) is a dual kinase inhibitor of epidermal growth factor receptor (EGFR) and human epidermal receptor two (HER-2), which is widely used in HER-2-positive breast cancer [14]. Furthermore, LAP sensitizes breast cancer cells to anticancer drugs by inhibiting the drug efflux function of ABC transporters [15]. 

In recent years, there are an increasing number of papers dealing with a lack of efficacy or the decreased efficacy of various drugs used in anticancer treatment. One of the frequently suggested strategies of overcoming this serious problem is the development of new drug dosage formulations which contain single drugs or their combinations. These novel drug delivery systems include polymeric nanoparticles as well as liposomes. The antitumor efficacy of such systems shows a cellular toxicity similar to that of the free compounds or can be markedly increased. PTX is one of the drugs that are most often incorporated into nanoparticles. Cosco et al. [16], using a PEGylated liposomal formulation containing PTX, showed that the encapsulation of the gemcitabine (GEM) in liposomes allowed an increase of its anticancer activity, while the PTX-loaded liposomes showed a cellular toxicity similar to that of the free compound. The contemporary presence of GEM did not induce destabilization in the liposomal structure and exhibited synergistic in vitro anticancer action between the co-encapsulated GEM and PTX. In addition, a multifunctional liposome used for the co-delivery of PTX and sorafenib was tested on the MCF-7 MDR breast cancer cell line. The liposomes were characterized by the ease of preparation, high loading capacity of drugs, good stability, high intracellular delivery and drug accumulation and caused higher cell apoptosis, the efficient reversal of MDR, and consequently increased cytotoxicity in tumor cells [17]. The increased anticancer efficacy and reversal of MDR in MCF-7 cells and MDR-resistant MCF-7 cells was also demonstrated by Huo et al. [18]. Their results showed that the disulfiram (DSF) and PTX co-loaded micelles exhibited effective cellular uptake and significantly increased the cytotoxicity of PTX to MCF-7/ADR cells, which could be due to the inhibitory effect of DSF on P-gp activity. Among the strategies developed to enhance drug delivery is the co-administration of P-gp inhibitors with an anticancer drug. A number of chemicals may be selected as the candidates, e.g., a newly synthesized tetraisohydroquinoline derivative (HZ08). This compound, loaded in a biomimetic nanovector, was used for delivery for MCF-7 breast cancer treatment. The increased cellular uptake of PTX and its prolonged intracellular retention due to the HZ08-mediated drug efflux inhibition was confirmed [19]. Polymer micelles characterize the core shell core-shell structure, where water insoluble drugs are encapsulated into the hydrophobic core and are surrounded by a water-soluble corona. Among various applications, these nanoparticles present chemotherapeutic potential in breast cancer. Polymer micelles may possess longer circulation times compared to surfactant micelles and accumulate in tumors through the enhanced permeability and retention (EPR) effect [20]. The other advantages associated with micellar drug delivery may be the limited side effects, enhanced therapeutic efficacy, and suitable pharmacokinetics. Additionally, polymer micelles can influence the response of target cells to overcome efflux-mediated MDR [21]. Therefore, polymeric micelles seem to be an appropriate choice for the delivery of chemotherapeutic agents in breast cancer.

In this study, we analyzed the cytotoxic effect of PTX and LAP on MCF-7 breast cancer cells, which are characterized by the absence of EGFR and HER-2 receptors. To find better strategies to overcome MDR, we analyzed the efficacy of PTX- and LAP-loaded micelles. It seems that such a micellar delivery system, which combines different strategies to overcome the efflux-mediated MDR, would be more effective for the treatment of resistant breast cancer than free PTX and LAP. Polylactide-co-poly(ethylene glycol) (PLA-PEG) was used to prepare micelles for the co-delivery of PTX and LAP. PEG is the most commonly used to form the hydrophilic shell of polymeric micelles, because it is biocompatible, reduces micelle aggregation and prevents interaction with serum proteins [22,23]. PLA is among the most common hydrophobic blocks that form the inner core due to its bioresorption and biocompatibility [24]. PLA-PEG micelles for single or multidrug delivery have been developed [25,26,27,28].

## 2. Materials and Methods

### 2.1. Materials 

Polylactide-co-poly(ethylene glycol) (PLA-PEG) diblock copolymer was synthesized according to the procedure described before [25]. The M_n_ of both the polylactide (PLA) and poly(ethylene glycol) (PEG) blocks was 5000. PTX was purchased from LC Laboratories (Woburn, MA, USA). LAP was purchased from Sigma-Aldrich (Poznan, Poland).

### 2.2. Preparation and Characterization of Micelles 

PLA-PEG micelles loaded with paclitaxel and lapatinib (PTX/LAP) were prepared by the co-solvent evaporation method. The ratio of drugs to polymer was 10% (w/w) and each drug was used in equal amounts. PLA-PEG with LAP was dissolved in methylene chloride, followed by addition of distilled water to obtain a concentration of 1 mg/mL. The mixture was stirred vigorously at room temperature for 3 h and left for solvent evaporation for 24 h. Then, PTX dissolved in ethanol was added to the micelle solution and the mixture was stirred for 3 h, followed by centrifugation at 12,300× *g* for 5 min (Eppendorf, rotor FA-45-6-30) to eliminate the unloaded drug. The supernatant was recovered, lyophilized and stored at 4 °C for further analysis. 

For the determination of the loading content (LC) and encapsulation efficiency (EE), lyophilized micelles were dissolved in acetonitrile and analyzed by high-performance liquid chromatography (HPLC, LaChrom Elite^®^VWR/Hitachi, Tokyo, Japan). Measurements were carried out with the use of a LiChroCART^®^ 150 × 4.6 mm Purospher^®^ STAR RP-18e (5 μm) column (Merck, Darmstadt, Germany) at a temperature of 25 °C at 227 nm. The mobile phase consisted of acetonitrile and water (70:30) at a flow rate of 0.5 mL/min.

The loading content was defined as the ratio of the weight of the drug to the weight of the drug-loaded micelles. The encapsulation efficiency was calculated as the ratio of the weight of the drug in the micelles to the weight of the drug added to the micelles.

Proton nuclear magnetic resonance (^1^H NMR) spectroscopy was performed on a Bruker-Avance II Ultrashiels Plus spectrometer (600MHz) using DMSO*-d_6_* as a solvent. Chemical shifts (δ) were given in ppm using tetramethylsilane as an internal reference. 

The morphology of the micelles was observed by transmission electron microscopy (TEM; Tecnai F20 TWIN microscope, FEI Company, Hillsboro, OR, USA. The microscope is equipped with a field emission gun (200 kV). Images were recorded on the Eagle 4k HS camera (FEI Company, Hillsboro, OR, USA) and processed with TIA software (FEI Company, Hillsboro, OR, USA). The micellar solution was filtered through a 0.85-μm filter and placed on a copper grid covered with carbon film and stained negatively (with the use of 2% phosphotungstic acid (PTA)) and air dried at room temperature before measurements. 

### 2.3. In Vitro Drug Release

The in vitro drug release was analyzed by the dialysis method with the use of 3-mL Slide-A-Lyzer dialysis cassette (Pierce; Rockford, IL, USA) with a MWCO of 3500 Da. Lyophilized drug-loaded micelles were dispersed (1 mg/mL) in pH 7.4 phosphate buffered saline (PBS) and added into a dialysis cassette. Each cassette was placed in 400 mL PBS (pH 7.4). A sample of 50 μL was drawn from each cassette at 1 h, 24 h, 48 h, etc. and replaced by the same volume of fresh PBS. At the same time, the PBS was changed to ensure sink conditions. The amount of the released drugs was measured by HPLC. DDsolver, an Excel Ad in Program [29], was used for modeling the kinetics of the dissolution processes by fitting the dissolution profiles with time-dependent equations. The fitting model is based on R^2^ (adjusted coefficient). 

### 2.4. Stability of Micelles 

The stability of PTX/LAP micelles (lyophilized or micelle solution) was analyzed for the percentage of initial drug loading and morphology. Micelles were stored at 4 °C for 6 weeks. For drug quantitation, the micellar solution was centrifuged at 12,300× *g* for 5 min and the supernatant was lyophilized, dissolved in acetonitrile and analyzed by means of HPLC. Lyophilized micelles were reconstituted in acetonitrile, filtered through a 0.45-µm filter and analyzed by HPLC. The morphology of micellar solution and lyophilized micelles reconstituted in water was observed by means of TEM.

### 2.5. Cell Culture

MCF-7 human epithelial breast cancer cells were obtained from the American Type Culture Collection (ATCC). MCF-7 were cultured in Eagle’s Minimum Essential Medium (EMEM) supplemented with 10% heat-inactivated fetal bovine serum (FBS; PAN-Biotech), 10 mM HEPES buffer (Sigma-Aldrich), 100 U/mL penicillin, 100 μg/mL streptomycin (Sigma-Aldrich), and 0.01 mg/mL human recombinant insulin (Sigma-Aldrich). Cells were maintained at 37 °C in a humidified atmosphere of 95% air and 5% CO_2_.

### 2.6. Cytotoxicity Assay

Free PTX and LAP stock solutions were prepared in DMSO and, as with the free micelles and drug-loaded micelles, were diluted with the appropriate volumes of the growth medium directly before the experiment. After 24 h of culture, the medium was replaced with the media containing PTX (25 nM), LAP (25 nM), the binary mixture of PTX and LAP, or PTX/LAP-loaded PLA-PEG micelles with the same concentration of the drugs. The initial cell density was 1 × 10^4^ cells/well and 96-well plates were used. The cultures were carried out for 24, 48, or 72 h. Control cells were incubated in the medium containing the non-toxic concentration of DMSO (v/v; 0.1%) or blank micelles in the same concentration as the experimental ones. At the end of the incubation period, the cytotoxicity was measured using a sulforhodamine assay according to the protocol described earlier [26]. Absorbance was measured at 570 nm and 690 nm (reference wavelength) using the MRX Revelation plate reader (Dynex Technologies).

### 2.7. P-gp Activity (Calcein-AM Assay)

P-gp activity was examined by determination of the intracellular accumulation of calcein, the fluorescent anionic dye, using the Multi-Drug Resistance Assay Kit (Calcein-AM; Cayman Chemical; USA) according to the manufacturer’s protocol. Calcein acetoxymethyl ester (calcein-AM) is a lipophilic, nonfluorescent dye that diffuses into cells where it is cleaved by cytosolic esterase to a hydrophilic and intensely green fluorescent calcein, which is well retained in the cytosol [30]. MCF-7 cells, at a density of 1 × 10^4^ cells/well in 100 μL of complete medium, were cultured in 96-well flat clear-bottom black-wall microplates for 24 h. Then, the cells were treated with PTX (25 nM), LAP (25 nM) or the binary mixture of PTX (25 nM) and LAP (25 nM) in DMSO or PTX, LAP or PTX/LAP-loaded PLA-PEG micelles at the same concentration for 1 h. After pretreatment with the compounds, 100 μL of the prepared calcein-AM solution was added to each well followed by incubation at 37 °C for another 30 min in the dark. Intracellular fluorescence was measured with a microplate reader (Triad LT Multimode Detector, Dynex Technologies) with an excitation wavelength of 485 nm and an emission wavelength of 535 nm. P-gp activity was expressed as a percentage of fluorescence measured in the treated wells relative to that in the untreated control wells. 

### 2.8. Statistical Analysis (In Vitro Studies)

Mean values ± standard deviations were calculated from the data obtained in 4 independent series of experiments. Statistica 10 PL software for Windows (StatSoft, Poland) was used to perform variance analysis (ANOVA) and Tukey’s HSD test. The p-value of less than 0.05 was considered significant. 

## 3. Results and Discussion

### 3.1. Characterization of Co-Loaded Micelles

**Figure 1:**
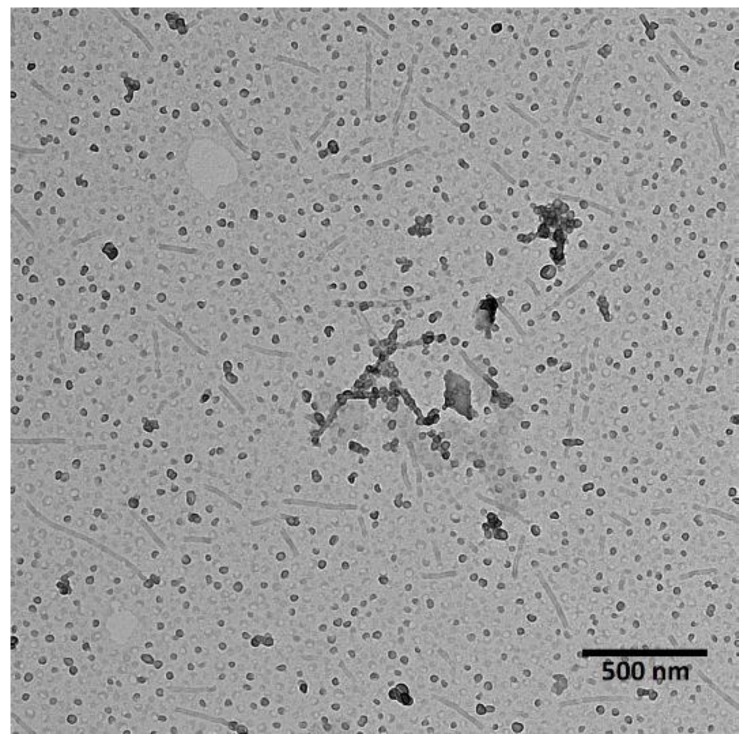
Transmission electron microscopy (TEM) image of polylactide-*co*-poly(ethylene glycol) (PLA-PEG) micelles loaded with paclitaxel (PTX) and lapatinib (LAP).

PLA-PEG was used to prepare micelles for the co-delivery of PTX and LAP. As shown in Figure 1, micelles of dual morphology were obtained—filomicelles of over 100 nm in length and spherical micelles of approximately 20 nm in diameter. The morphology of polymeric micelles greatly influences their circulation time, biodistribution, cellular uptake, intracellular trafficking, and overall efficiency. Nanocarriers with dual morphology could be promising for pharmaceutical applications. Spherical micelles, which characterize more rapid drug release, provide a high initial drug dose, which is maintained by slow-releasing filomicelles. This is a novel approach, because so far only spherical PLA-PEG micelles were developed for delivery of PTX and LAP [31].

**Figure 2:**
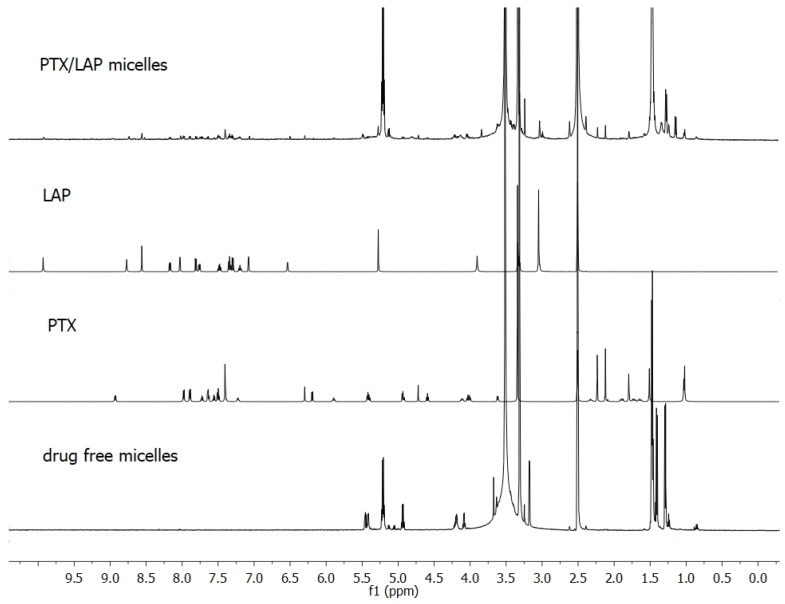
Proton nuclear magnetic resonance (^1^H NMR) spectra of PTX, LAP, drug-free PLA-PEG micelles, and drug-loaded PLA-PEG micelles in deuterated dimethyl sulfoxide (DMSO*-d_6_*).

The effective incorporation of PTX and LAP into micelles was shown by means of NMR analysis. Figure 2 presents ^1^H NMR spectra of individual drugs, lyophilized drug-free micelles and lyophilized drug-loaded micelles. Signals arising from the methine (5.2 ppm) and methyl (1.5–1.4 ppm) protons of PLA and methylene protons of PEG (3.6 ppm) were observed in the drug-free micelles. In the spectrum of PTX/LAP-loaded micelles, signals arising from the drugs were detected in addition to all the signals of the copolymer. PTX was identified by proton resonances at 1.0 ppm assigned to its methyl groups at C16 and C17 and LAP by signals at 8.6 and 6.5 ppm belonging to the aromatic groups. As shown in Table 1, PTX displays higher loading capacity (2.47%) compared to LAP (1.62%). This finding can be explained by interactions between OH and NH groups of PTX with the C=O group of PLA [27,32]. Intermolecular interactions may facilitate better drug entrapment inside the micelles. Similar results were obtained with other multidrug micelles containing PTX (e.g., PTX and 17-*N*-allylamino-17-demethoxygeldanamycin) [27].

### 3.2. Drug Release Study

**Figure 3:**
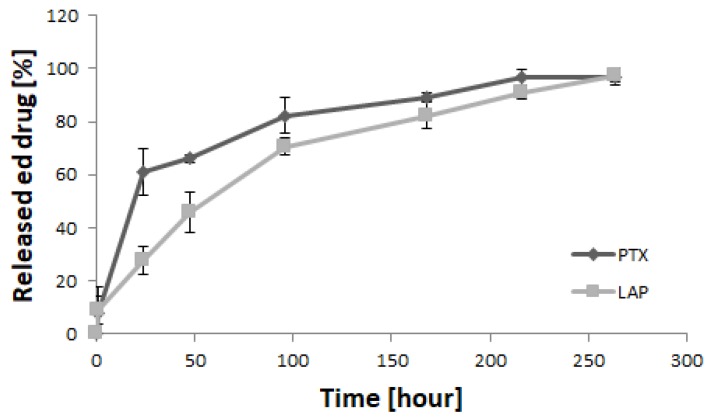
In vitro release of drugs from PTX/LAP-loaded PLA-PEG micelles (*n* = 3; ±SD).

The release of drugs from the micellar system was studied under in vitro conditions (Figure 3). Similar amounts of both drugs were released from the micelles after 1 h (8.0% of PTX and 8.7% of LAP). However, PTX exhibited higher release after 24 h (61.2%) compared to LAP (27.6%). Beyond this, the release of PTX slowed down and was almost completed after 264 h (97%). Contrary to PTX, the release of LAP gradually increased to 45.9% after 48 h, 70.7% after 96 h and 97.5% after 264 h. 

In order to determine the mechanism of drug release from the micelles, the release data were fitted to Peppas–Sahlin kinetic model, which is described by the following expression: M_t_/M_∞_ = k_1_t*^m^* ^+^ k_2_t^2*m*^ (where k_1_ and k_2_ are the Fickian kinetic constant and the erosion rate constant, respectively). The coefficient *m* is the purely Fickian diffusion exponent [33]. The Peppas–Sahlin model is used when the release mechanism is unknown or when several types of release phenomena could be involved [34]. The release data of PTX and LAP fitted well with the Peppas–Sahlin model (R^2^_adjusted_ = 1.00 and R^2^_adjusted_ = 0.99 for PTX and LAP, respectively). LAP was mostly released by Fickian diffusion (*m* = 0.42) [33]. The higher value of coefficient *m* for PTX (*m* = 7.62) indicates anomalous transport [33]. The higher initial release of PTX was probably caused by diffusion, which proceeded faster due to a higher molar mass and higher loading content compared to LAP. Beyond this, the release process of PTX was controlled by copolymer degradation that proceeds slowly for longer PLA blocks and drug–polymer interactions [26,27].

### 3.3. Stability of PLA-PEG Micelles

**Figure 4:**
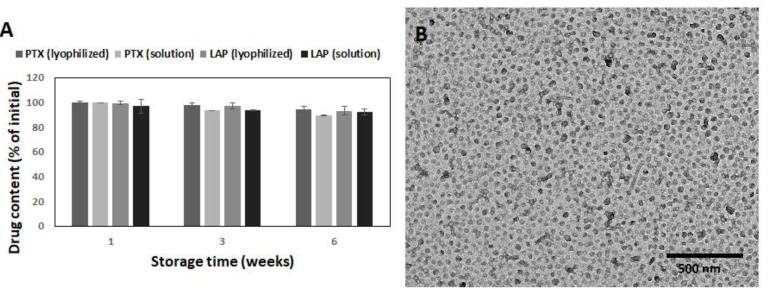
Stability of the PTX/LAP-loaded micelles during storage: PTX content (**A**) and morphology of micellar solution after 6 weeks (**B**). SD shown as error bars, *n* = 3.

To determine the stability of the drug-loaded micelles, the drug content and morphology was analyzed (Figure 4). Lyophilized micelles retained both the initial drug content and morphology for at least 6 weeks. Thus, it was confirmed that the lyophilization process does not compromise micellar properties. Some changes in the morphology of the micelles stored in solution were observed, because spherical nanoparticles were the vast majority after 6 weeks (Figure 4B). The same effect caused by hydrolytic degradation was also observed for other bioresorbable polymers, e.g., poly(ε-caprolactone)-*co*-PEG [35]. The PTX and LAP contents in micellar solution decreased insignificantly to 93.4% and 93.5% after 3 weeks and to 89.5% and 92.4% after 6 weeks, respectively (Figure 4A). The data indicated great stability of the PTX/LAP-loaded micelles in both the lyophilized and solution forms. 

### 3.4. Effect of PTX and LAP on MCF-7 Breast Cancer Cells

**Figure 5:**
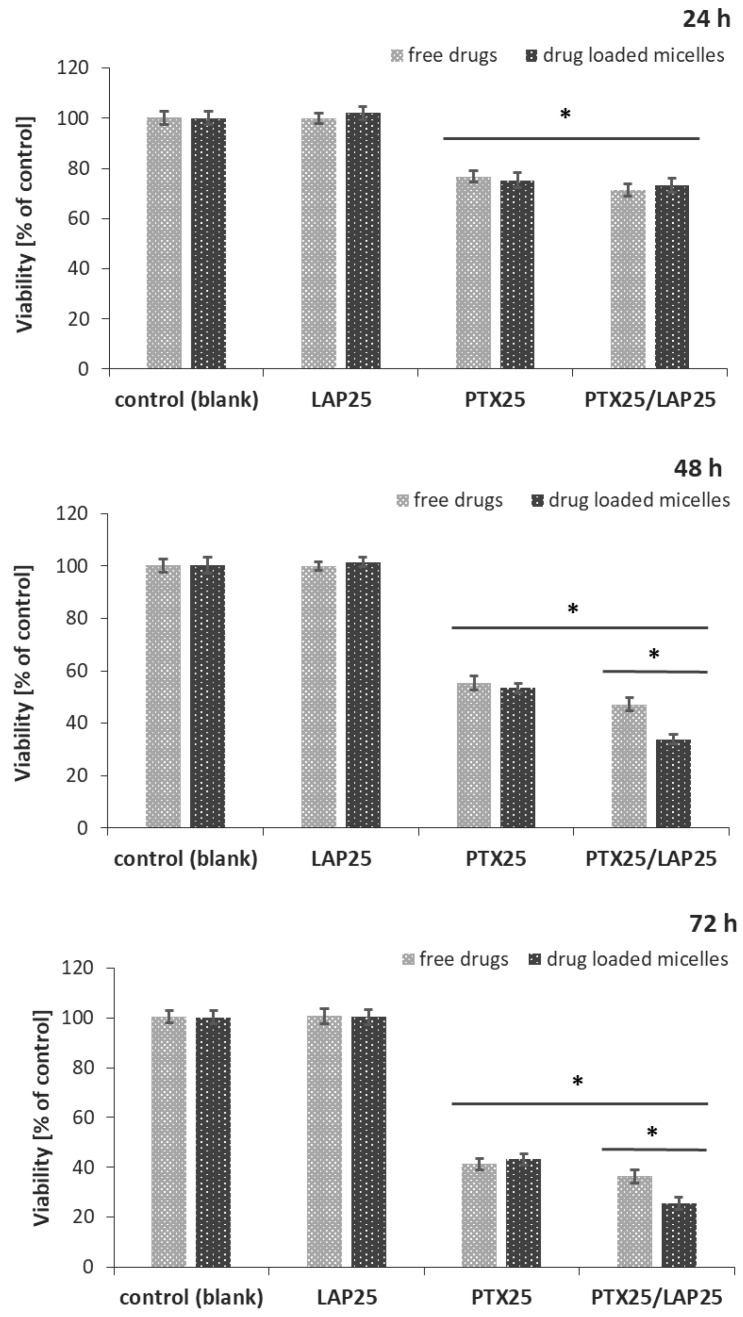
Cytotoxic effect of the PTX (25 nM) and LAP (25 nM) combination on MCF-7 breast cancer cells. MCF-7 cells were treated with PTX-, LAP- and PTX/LAP-loaded micelles, and the mixture of free drugs for 24 h, 48 h or 72 h and then cell viability was determined by sulforhodamine B assay and expressed as a percentage of the control (mean ± SD, *n* =4). * *p* < 0.05 vs. control; PTX/LAP-loaded micelles vs. free PTX/LAP.

The cytotoxic effects of the blank micelles, PTX-, LAP-, and PTX/LAP-loaded PLA-PEG micelles were tested in vitro on MCF-7 breast cancer cells. A comparison was made with a mixture of free drugs in the same concentrations as in the drug-loaded micelles. The MCF-7 cell line, derived from the mammary glands’ metastatic sites, was chosen as a model for the study because of the lack of EGFR and HER-2 receptors which implies its insensitivity to LAP. As shown in Figure 5, the cell viability was significantly decreased by both the PTX- and PTX/LAP-loaded micelles and the mixture of free drugs. The PTX-loaded micelles exhibited comparable cytotoxicity to cells as the free drug. However, the PTX/LAP-loaded micelles showed greater toxicity compared to the binary mixture of PTX and LAP after 48 h and 72 h; the toxicity of the PTX/LAP dual-loaded micelles increases with time, which suggests that this effect is achieved by simultaneous transport of both active agents into cells and their sustained release. Importantly, the blank micelles, LAP-loaded micelles, and free LAP did not affect cell proliferation, indicating that the cytotoxicity of PTX-, PTX/LAP-loaded micelles, and the binary mixture of free PTX and LAP was solely caused by PTX. Therefore, dual drug-loaded micelles could enhance the cytotoxicity of PTX against breast cancer cells. These findings are in agreement with the data describing the effect of paclitaxel–lapatinib-loaded Pluronic F127 micelles. Although HER-2-positive SKBR3 breast cancer cells were used [36], the authors found that LAP and pluronic sensitized the cancerous cells to PTX via efflux pump inhibition. The micellar system was passively targeted by the enhanced permeability and retention effect. The MTT (3-[4,5-dimethylthiazol-2-yl]-2,5 diphenyl tetrazolium bromide) assay demonstrated that the drug-loaded micelles had greater cytotoxicity compared with the free drugs. Wei et al. [31] used a micellar system of LAP and PTX to investigate the influence of both drugs on SKBR3 (HER-2-positive) and MDA-MB-231 (HER-2-negative) cells. The authors showed that the cytotoxicity of PTX/LAP micelles against SKBR3 cells significantly increased as compared with PTX micelles, whereas there was no significant difference against MDA-MB-231 cells. Thus PTX/LAP micelles could be a promising drug delivery system for HER-2-positive breast cancer. In another study, PTX and LAP-loaded lipopolymer micelles (poly(ethylene glycol)-block-poly(2-methyl-2-carboxylpropylene carbonate-graftdodecanol)) were used to determine whether the combination therapy with these two drugs could synergistically influence resistant prostate cancer. It was found that co-treatment of the cells resulted in the increased toxicity and a marked MDR reversal compared to PTX alone [37].

### 3.5. P-gp Activity in MCF-7 Breast Cancer Cells

To find out whether free LAP and drug-loaded PLA-PEG micelles overcome MDR in MCF-7 breast cancer cells, calcein-AM assay was performed to determine their effect on P-gp activity. The kit contained a cell-permeable nonfluorescent P-gp substrate (calcein-AM), which is cleaved intracellularly into a fluorescent molecule (calcein) for the detection of P-gp activity.

**Figure 6:**
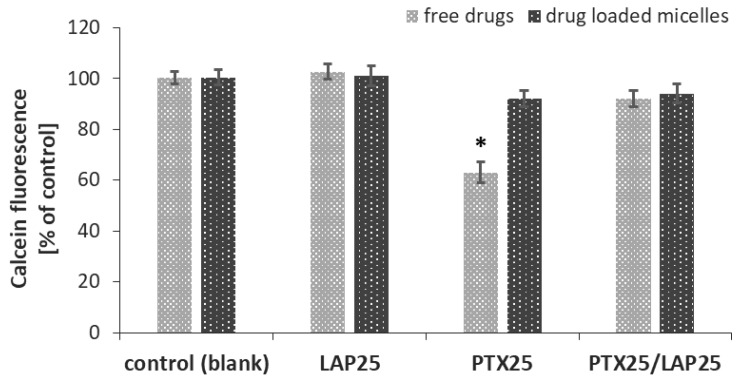
Effect of the PTX (25 nM) and LAP (25 nM) combination on P-gp activity in MCF-7 breast cancer cells. MCF-7 cells were treated with PTX-, LAP- or PTX/LAP-loaded micelles, and the mixture of free drugs for 1 h and then calcein-AM assay was performed. Calcein fluorescence was expressed as a percentage of the control (mean ± SD, *n* = 4). * *p* < 0.05 vs. control.

As shown in Figure 6, only free PTX induced P-gp activity and led to a decrease in intracellular calcein fluorescence, while the free LAP, the mixture of free LAP and PTX, blank micelles as well as micelles containing either LAP or PTX alone or a combination of these two drugs did not influence MDR. PTX-loaded micelles did not enhance P-gp activity, while LAP suppressed P-gp activity in MCF-7 cells induced by free PTX.

It is known from several in vitro and in vivo studies that LAP not only effectively inhibits the proliferation of HER-2-positive breast cancer but also strongly sensitizes PTX-resistant tumors to PTX [31]. Co-treatment with LAP and PTX shows synergistic effects as compared with PTX alone for patients with HER-2-positive breast cancer [38]. In this study, we have shown that the combination of PTX with LAP, in particular when loaded into micelles, can reduce the viability of HER-2-negative breast cancer cells. This effect is potentiated by LAP and involves MDR suppression. 

## 4. Conclusions

PLA-PEG bioresorbable micelles present many advantages as drug delivery systems, e.g., biocompatibility and sustained drug release. This study aimed to develop a novel micellar carrier for the co-delivery of PTX and LAP. The simultaneous incorporation of both drugs into PLA-PEG micelles was confirmed by ^1^H NMR. TEM analysis revealed the dual morphology of micelles, which is advantageous for providing a higher initial dose of the active agent by spherical micelles and the prolonged release by filomicelles. In fact, the micelles provided the release of PTX and LAP for over 264 h. The PTX/LAP-loaded PLA-PEG micelles (lyophilized and solution) showed high stability during storage at 4°C for at least 6 weeks. Drug-free micelles did not affect the viability of cells. Moreover, this study showed the feasibility of using a LAP and PTX combination to overcome MDR in MCF-7 cells. It is concluded that PTX/LAP micelles could be promising for uses not only in the therapy of HER-2-positive, but also HER-2-negative breast cancers.

## Figures and Tables

**Table 1:** Drug loading and encapsulation efficiency data of PLA-PEG micelles (± SD, *n* = 3).

Drug	LC [%]	EE [%]
Paclitaxel	2.5 ± 0.3	49.4 ± 3.1
Lapatinib	1.6 ± 0.2	32.4 ± 3.5
